# Springback characteristics and influencing laws of four-axis flexible roll bending forming for aluminum alloy

**DOI:** 10.1371/journal.pone.0306604

**Published:** 2024-08-27

**Authors:** Peng Chen, Shihong Lu

**Affiliations:** College of Mechanic and Electrical Engineering, Nanjing University of Aeronautics and Astronautics, Nanjing, China; University of Sharjah, UNITED ARAB EMIRATES

## Abstract

This study aims to solve the problem of springback control of aluminum alloy components in the rolling process, and the method of combining experiment and simulation is adopted. Firstly, a series of aluminum alloy samples are designed, and the four-axis flexible bending machine is used for precision roll bending. Secondly, the three-dimensional (3D) shape change data of the workpiece before and after roll bending is monitored and recorded in real-time by a high-precision 3D scanner. Meanwhile, aiming at different rolling process parameters of each group (including roll bend speed, feed rate, pre-deformation amount, mold curvature radius, and other factors), advanced finite element software is used to carry out detailed simulation and calculations. In addition, the coincidence is compared and analyzed between the actual experiment results and the simulation prediction. The stress-strain distribution and springback evolution of aluminum alloy during roll bending are described accurately. The experimental and simulation results show that the springback rate of aluminum alloy fluctuates in the range of 5% to 15% after four-axis flexible roll bending, and the specific springback value is influenced by various process parameters. For example, under the premise of keeping other conditions unchanged, when the roll bending speed is increased from 30mm/s to 60mm/s, the springback rate shows an upward trend of about 3%. By increasing the feed rate by 20%, an average decrease of about 7% in springback quantity is observed. It can be seen that the increase in roll bending speed can aggravate the springback phenomenon, and the appropriate increase in feed rate can play a certain role in restraining the springback. Further analysis shows that the choice of the mold curvature radius and pre-deformation amount also has a decisive influence on the springback characteristics. There is a nonlinear relationship between the two parameters and the amount of springback. Changing these two parameters in a specific range can effectively regulate the springback effect.

## 1. Introduction

In today’s world, with the swift growth of industrial technology, aluminum alloy has become an indispensable material in aerospace, automobile manufacturing, high-speed train, and shipbuilding industries due to its unique physical and chemical properties, such as lightweight, high strength, good corrosion resistance, and formability [1–3]. Especially in the context of automotive lightweight, the application of aluminum alloy is expected to further expand to reduce energy consumption and emissions [4,5]. However, in the process of aluminum alloy processing, especially in the complex mechanical process of roll bending forming, springback phenomenon has become a major problem affecting the dimensional accuracy and shape quality of the finished product [6,7].

Roll bending forming is an efficient sheet metal forming process that produces complex curved shapes through continuous bending [8]. At the same time, springback, as a material elastic recovery phenomenon, is particularly evident after the end of the rolling forming process. It leads to dimensional deviations between the formed part and the design model, which are often intolerable in the precision manufacturing field [9–11]. Therefore, controlling and predicting the springback phenomenon in aluminum alloy roll bending forming is of great practical significance for improving product quality, reducing rework, and reducing cost [12].

Domestic and international scholars have extensively studied the springback phenomenon of metal materials. Most of these studies focus on the mechanism analysis of springback, the exploration of influencing factors, and the establishment of prediction models, etc., affording the theoretical basis and technical approach for springback control [13–15]. However, given the special properties of aluminum alloy materials and the process complexity of the four-axis flexible roll bending forming, the existing research results still face certain limitations in practical applications [16]. Especially for the advanced forming technology of four-axis flexible roll bending, how to accurately control and predict the springback phenomenon is still an urgent technical problem to be solved [17–19].

Based on this, this study aims to deeply explore the springback characteristics of four-axis flexible roll bending forming for aluminum alloy and its influence rule. Through the combination of experimental research and advanced simulation, this study reveals the stress-strain distribution and springback evolution of aluminum alloy during the four-axis flexible roll bending. Furthermore, this study also explores the key process parameters affecting springback, offering a scientific basis and technical guidance for the precise control of springback in aluminum alloy roll bending forming. This study has remarkable innovation in the springback characteristics and its influence law of aluminum alloy four-axis flexible roll bending. Firstly, the springback phenomenon of aluminum alloy during roll bending is comprehensively analyzed by combining experiment with finite element simulation. Secondly, the study not only discusses the influence of single parameter on springback, but also deeply analyzes the springback characteristics under the comprehensive action of multiple process parameters, which provides a theoretical basis for springback control under complex process conditions. In addition, this study also pays special attention to the nonlinear influence of the two key parameters, the radius of curvature and the pre-deformation, on the springback characteristics, revealing its internal law, which provides a new perspective for the optimization of aluminum alloy roll bending forming process. Finally, the high-precision simulation prediction and experimental verification of the research results provide practical technical support for the quality control and cost reduction of aluminum alloy forming parts in industrial production, which has important engineering application value.

## 2 Literature review

As a kind of lightweight and high-strength engineering material, the mechanical properties and forming process of aluminum alloy received extensive attention. Qin & Chen (2022) [20] argued that the mechanical properties of aluminum alloy, including elastic modulus, yield strength, and plastic deformation ability, had a decisive influence on its behavior in the forming process. In particular, Akopyan et al. (2020) [21] believed that in the complex mechanical process of roll bending forming, these mechanical properties were closely related to the microstructure of materials, determining the formability and finished product quality of aluminum alloys. In addition, Wang et al. (2021) [22] pointed out that the microstructure characteristics of different aluminum alloys, such as grain structure and grain boundary distribution, significantly affected their mechanical behavior and roll bending forming properties.

In the roll bending forming domain, the springback phenomenon was one of the research hotspots. Early studies by Mulidran et al. (2018) [23] mainly concentrated on the establishment of an analytical model of springback, trying to predict the amount of springback through a simplified physical model. With the development of computer technology, in recent years, more and more researchers have begun to use finite element analysis (FEA) technology to simulate roll bending forming, thus predicting springback phenomenon more accurately [24]. For example, Gavrilescu et al. (2021) [25] proposed a hybrid numerical analysis method to study the three-rolling process of two steel plates used in the naval industry. The FEA method was employed to model the bending process, and the regression model of the bending force as a function of plate thickness and vertical displacement of the upper roll was established. These studies not only involved the springback characteristics of different materials but also discussed the influence of process parameters such as roll bending radius and speed on springback. Meanwhile, recent studies by Izadpanah & Amini (2023) [26] also found the influence of strain hardening behavior of various materials on springback characteristics during roll bending forming, thereby deepening the understanding of springback phenomenon. Li et al. (2023) [27] compared the heat transfer characteristics of single-phase convection, subcooled boiling, and saturated boiling under rolling and vertical conditions. Wang et al. (2016) [28] studied the influence of particle size and porosity on permeability.

Although previous studies made some progress in understanding and controlling the springback phenomenon, there were still several problems. Firstly, most studies focused on the impact of a single factor on springback, and there was a lack of explorations under the combined action of multiple parameters [29]. Secondly, for the special forming process of four-axis flexible roll bending, the existing springback prediction model was difficult to directly apply, and further research was needed [30]. Finally, the influence mechanism of the microstructure of aluminum alloy on its springback behavior was not been fully explored, which limited the in-depth understanding of springback phenomenon [31,32].

To solve the above problems, this study focused on exploring the influencing factors of springback phenomenon in four-axis flexible roll bending for aluminum alloy, especially the springback characteristics under the combined action of multiple process parameters. Through the establishment of more complex FEA models, combined with experimental verification, the purpose of this study was to deeply analyze the comprehensive influence of process parameters such as pre-deformation amount, roll bending speed, feed rate, and mold curvature radius on springback quantity. In addition, the influence mechanism of microstructure change of aluminum alloy on springback phenomenon was also discussed in this study, to provide a more scientific theoretical basis and technical guidance for accurate control of springback phenomenon in aluminum alloy roll bending forming. By filling these research gaps, this study is expected to contribute to the development of aluminum alloy roll bending forming technology.

## 3 Research methodology

### 3.1 Experimental materials and sample preparation

To prepare the experimental sample, the high-performance 7075 aluminum alloy is selected as the raw material. The material is widely used in the aerospace field because of its excellent mechanical properties and outstanding formability. The main chemical composition of 7075 aluminum alloy includes aluminum (Al), zinc (Zn), magnesium (Mg), copper (Cu), etc., of which Zn is the main alloying element in the alloy system, giving the material good mechanical properties. The specific chemical composition and basic mechanical property parameters are exhibited in Table 1:

**Table 1:** Chemical composition and performance parameters of 7075 aluminum alloy.

Category	Parameter	Value
Chemical composition	Aluminum (Al)	Allowance
	Zinc (Zn)	5.1–6.1
	Magnesium (Mg)	2.1–2.9
	Copper (Cu)	1.2–2.0
	Others	<0.5
Mechanical performance parameters	Yield strength (MPa)	505
	Tensile strength (MPa)	572
	Elongation rate (%)	11

Table 1 lists the chemical composition and basic mechanical properties of 7075 aluminum alloy in detail, which makes it a widely used material in aerospace field. 7075 aluminum alloy takes Aluminum (Al) as the base metal, supplemented by alloying elements such as Zinc (Zn), Magnesium (Mg) and Copper (Cu), among which zinc is the main alloying element in the alloy system, which provides good mechanical properties for the material. Specifically, zinc content ranges from 5.1% to 6.1%, magnesium content ranges from 2.1% to 2.9%, copper content ranges from 1.2% to 2.0%, and the total content of other elements is less than 0.5%.

**Fig 1:**
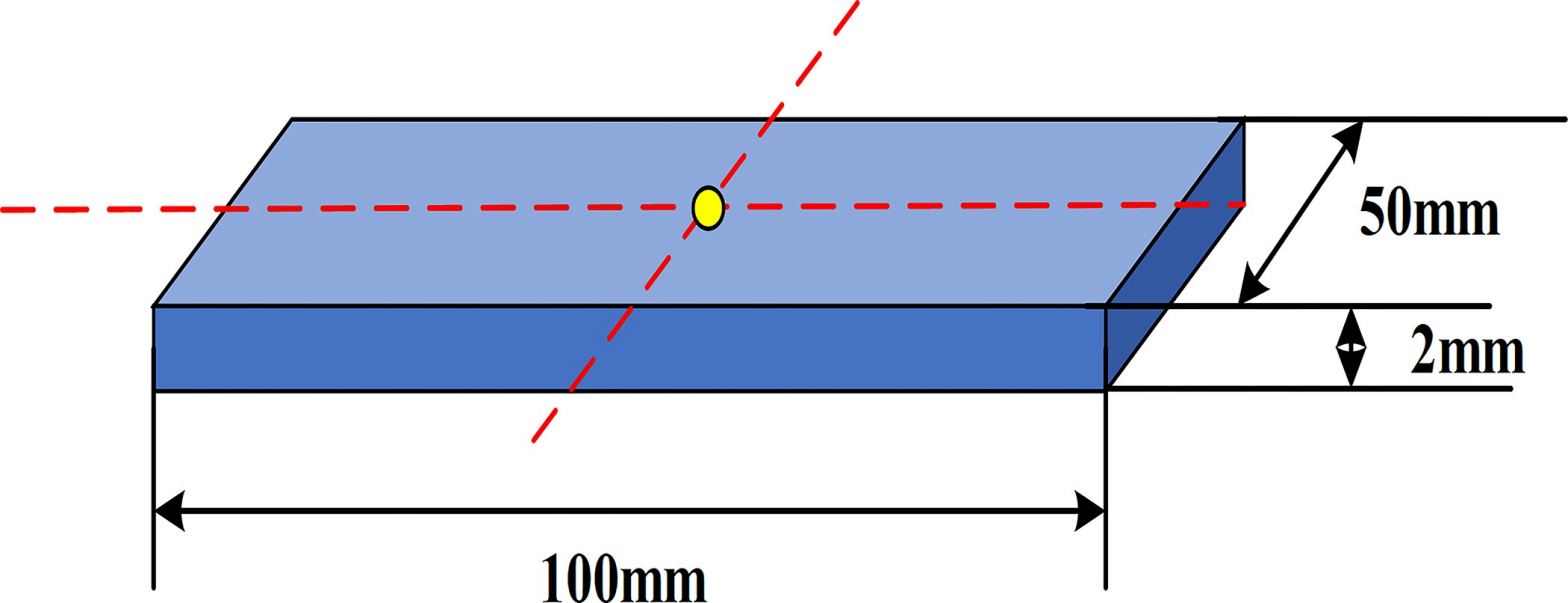
7075 aluminum alloy sample size.

In terms of mechanical properties, 7075 aluminum alloy shows excellent properties, with yield strength of 505 MPa, tensile strength of 572 MPa and elongation of 11%. These data show that 7075 aluminum alloy not only has high strength and hardness, but also maintains certain plasticity and toughness. This balanced combination of mechanical properties makes 7075 aluminum alloy very suitable for manufacturing aerospace components requiring high strength and good formability. Therefore, choosing 7075 aluminum alloy as experimental material can ensure the scientific results and the reliability of practical application. To more intuitively understand these samples’ specific size and shape, a detailed schematic diagram is provided in Fig 1.

Fig 1 provides a schematic diagram of the specific dimensions of the 7075 aluminum alloy sample, which is very important for understanding the geometric characteristics of the experimental sample. The sample is designed in a rectangular size of 100mm x 50mm x 2mm, aiming at simulating the common dimensions of aluminum alloy parts in practical industrial applications. This size design helps to ensure the applicability of the research results in wider industrial applications. Reference points for alignment and measurement are also marked in Fig 1, which play a key role in the experimental process, especially in the accurate three-dimensional deformation measurement. These preset marking points ensure that the change of sample shape can be accurately recorded and analyzed before and after rolling bending. In addition, the surfaces of all samples have been carefully polished to eliminate microscopic defects that may be introduced during cutting. First, rough sandpaper (P400) is used for preliminary grinding to remove burrs and uneven surface. Then gradually transition to fine sandpaper (P800 to P2000) for fine grinding. Finally, polishing paste is used to polish the surface of the sample to achieve the mirror effect. Reference points are separately marked at the front and rear ends of the sample to measure the shape change after roll bending. In addition, high-precision digital calipers are used to measure and record the initial size of each sample to ensure the accuracy of subsequent data analysis.

After the above steps are completed, all samples are kept in a dry and dust-free environment to prevent surface oxidation or contamination until the roll bending forming experiment begins. In addition, to ensure the repeatability of the experiment, at least three identical samples are prepared for each experimental condition.

### 3.2 Four-axis flexible roll bending experiments

According to the samples prepared above, the four-axis flexible roll bending experiments are carried out. The core equipment of this experiment is a four-axis flexible roll bending machine equipped with a high-precision control system, which is designed to achieve accurate roll bending of aluminum alloy sheets. The machine’s control system is based on the latest microprocessor technology and enables real-time monitoring and adjustment of key parameters during the machining process to ensure machining accuracy and repeatability.

The following are the main technical parameters of the roll bending machine: Roller diameter: 200 mm. This diameter selection allows for versatility and flexibility, allowing the machine to adapt to the processing requirements of aluminum alloy sheets with different thicknesses and widths. Max roll bending speed: 100 mm/s. This upper-speed limit ensures processing efficiency while meeting the need to control the stress state of the material during the rolling process by adjusting the speed, thereby affecting springback characteristics. The adjustment range of the feed rate: 0 to 50 mm. This adjustable range of the feed rate allows the experimenter to precisely control the rate at which the material moves under the roller depending on the specific roll bending task and aluminum material characteristics. It is critical for controlling the forming accuracy and springback phenomenon. Roller pressure adjustment range: 0 to 10 kN. By adjusting the pressure of the roller on the material, the plastic deformation behavior of the material during the rolling process can be further fine-tuned to optimize the forming effect and reduce springback.

The control of environmental conditions in the experiment is also one of the key factors for the experiment’s success. All roll bending experiments are performed at constant room temperature to exclude possible effects of temperature changes on the results. Processing at room temperature means that all experimental data can be collected and compared under a unified benchmark, thus improving the reliability and consistency of experimental results.

Then, the specific process of the experiment is carried out. First, the 7075 aluminum alloy sample is carefully prepared according to the specific dimensions shown in Fig 1. The samples are all cut from the same aluminum alloy sheet using high-precision wire-cutting technology to ensure consistency in material and initial state. The size of each sample is strictly in accordance with the design requirements, guaranteeing the standardization and repeatability of the experiment. Before the roll bending machine experiment began, the process parameters of the four-axis flexible roll bending machine were carefully set according to the pre-designed experiment scheme. These parameters include, but are not limited to, roll bending speed and feed rate, which are adjusted by the machine’s high-precision control system. Each set of parameters is designed to explore its specific influence on the roll bending forming effect and springback characteristics of aluminum alloy.

After setting the parameters, the 7075 aluminum alloy sample is precisely placed in the machining position of the roll bending machine. Subsequently, the roll bending machine is started and the roll bending operation begins according to the predetermined parameters. To ensure the reliability and accuracy of the experimental data, each sample is subjected to three repeated roll bending operations, with a recovery period after each operation to eliminate possible internal stress effects. Immediately after each roll bending, the aluminum alloy sample is measured using an advanced 3D scanner. The purpose of this step is to accurately document the 3D shape change of the sample after roll bending, with particular attention to the geometry of its bent part, for subsequent analysis of the springback phenomenon.

This study particularly concerns the effects of roll bending speed and feed rate on the springback characteristics of aluminum alloy. For this purpose, the following parameter range is designed for the experiment: roll bending speed set at seven different speed levels, from 30 mm/s to 60 mm/s. Such a design aims to investigate the effect of velocity changes on the stress state of the material and its subsequent springback behavior. The feed rate is also set at three levels, 10 mm, 15 mm, and 20 mm. The variation of feed rate directly affects the material’s deformation degree during processing, which may affect the amount of springback after roll bending.

### 3.3 Finite element simulation

A specific process of finite element simulation is adopted to further validate and refine the experimental results theoretically and provide a more in-depth understanding of the stress-strain behavior of aluminum alloy in the complex rolling process and its influence on the springback phenomenon. A detailed aluminum alloy roll bending forming model is constructed in professional FEA software. The model is based on the same 7075 aluminum alloy material properties used in previous experiments. These properties, including but not limited to elastic modulus, yield strength, Poisson’s ratio, etc., are based on the specific test data of experimental materials in the previous stage. Precise loading and boundary conditions are also set in the model, which strictly corresponds to the actual rolling process parameters, such as roll bending speed, feed rate, etc., to guarantee the practicality and accuracy of the simulation.

**Fig 2:**
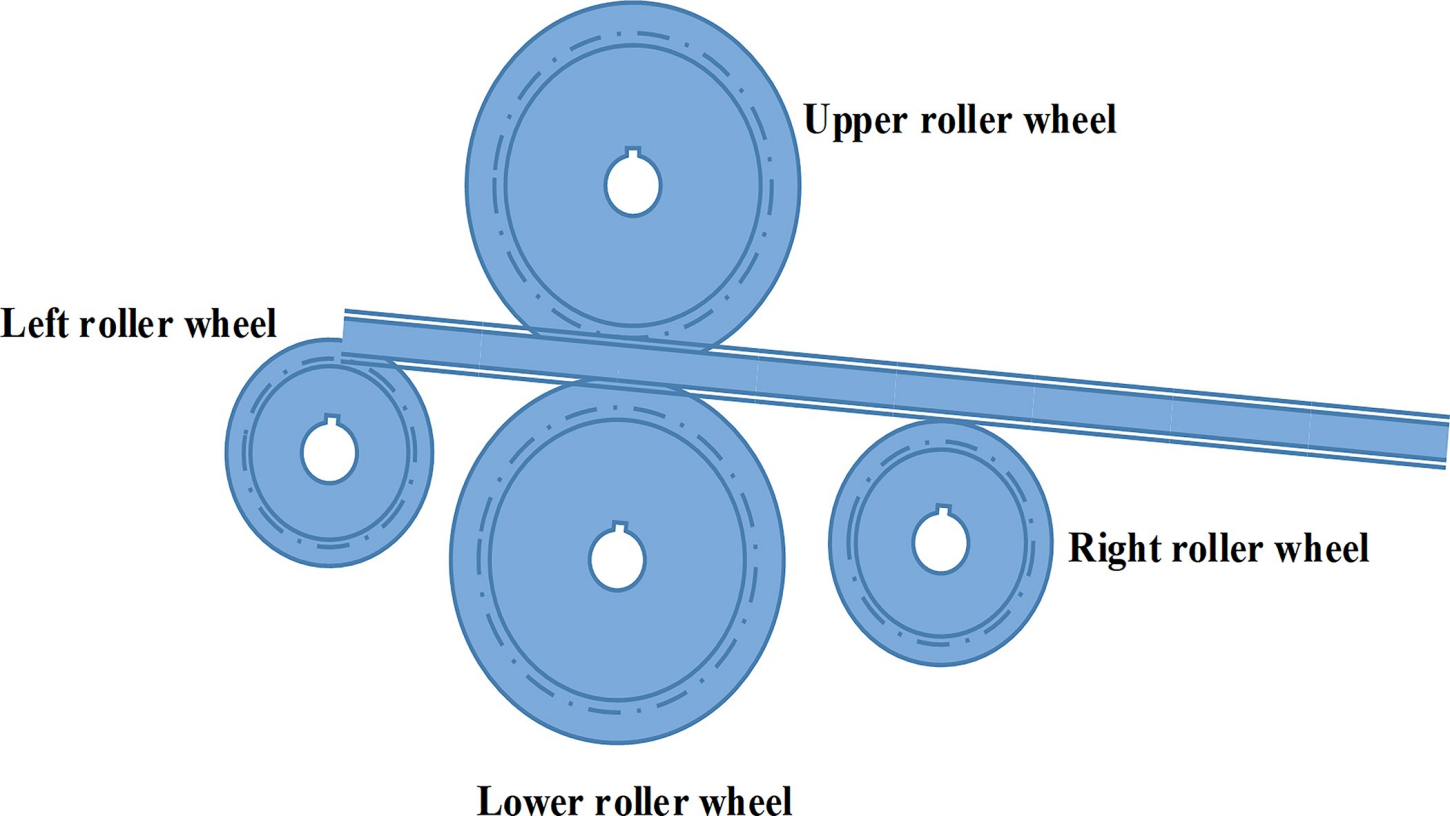
Roll bending finite element simulation model of L profile.

When constructing the finite element simulation of the rolling process, the L-section-based method is adopted to form the model core. The roller wheel in the simulation is treated as a discrete rigid body element to ensure its fixed and invariant properties during the rolling process. In contrast, the L profile is endowed with the properties of a deformable shape, so that it can show the bending and deformation of the material in the simulation process. This setup makes for an intuitive view of the roll bending finite element model, as depicted in Fig 2.

**Fig 3:**
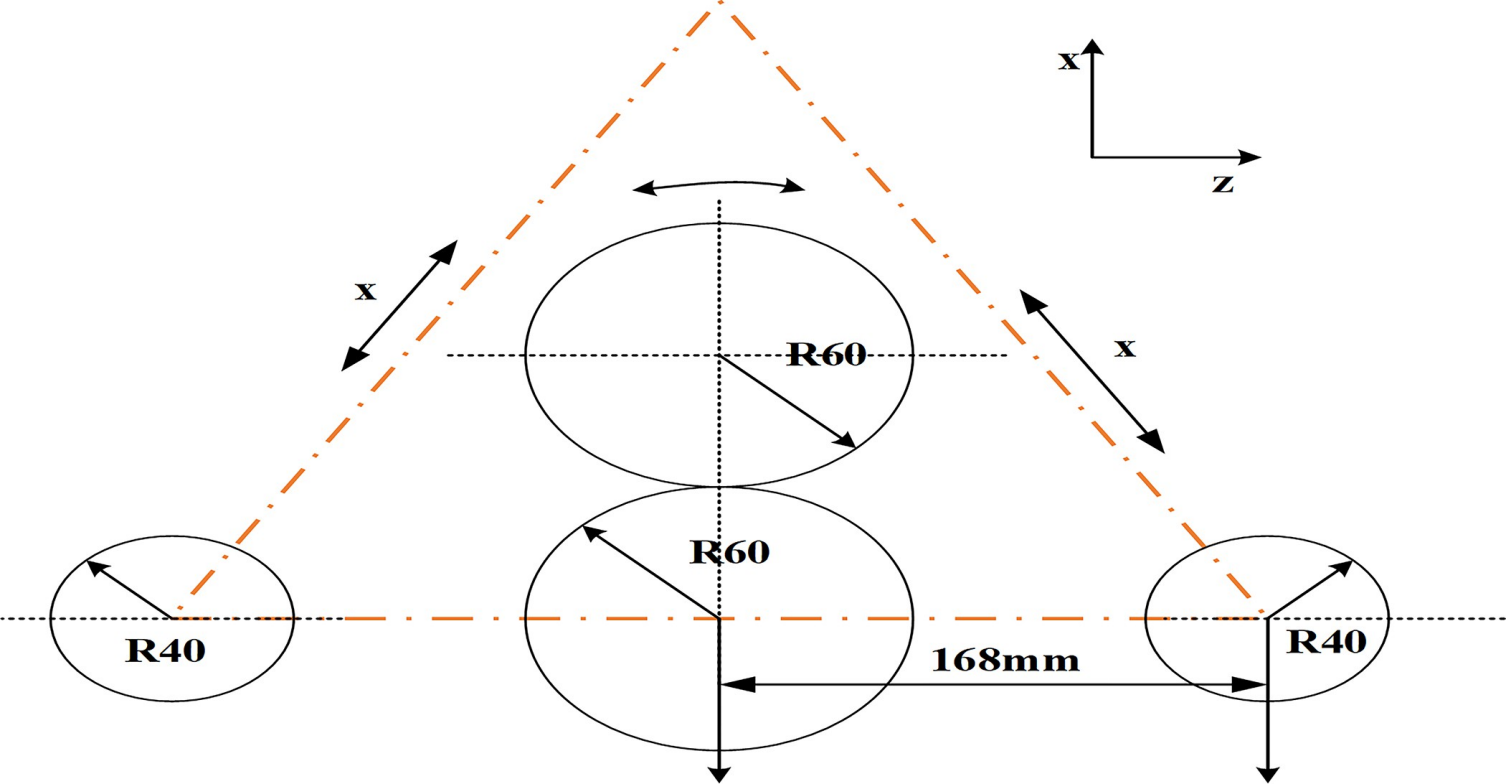
L profile’s roll bending simulation model.

Then, according to the four-axis roll bending principle, as shown in Fig 3, the specific operation steps are determined. Moreover, the analysis step is carefully set, a total of four steps, to ensure a high degree of consistency between the simulation process and the actual roll bending operation.

Fig 3 shows the roll-bending simulation model of L-shaped section, and the dimension information such as "R60", "R40" and "168mm" is marked. Here, "R" stands for radius, so "R60" and "R40" refer to the radius of the roller or the radius of curvature of the material contact point during rolling and bending. "168mm" refers to the diameter of the roller or the size of a specific part during rolling and bending. Arrows are used in Fig 3 to indicate the position change of the roller during rolling and bending, which helps the observer to understand how the roller moves along the surface of the material. In addition, the arrow can also indicate the bending direction of the material in the process of roll bending, that is, how the material is deformed under the action of the roller.

1) Left-axle pre-bending: In the initial stage of the roll bending, the left roller is first started to pre-bend. This step is mainly to create an initial bend at one end of the material. This stage is crucial because it sets the starting point for the entire rolling process, ensuring that the material deforms in a predetermined path during subsequent operations.

2) Right-axle pre-bending: Immediately after the left axle pre-bending, the right roller is also pre-bending, to form a corresponding initial bend at the other end of the material. This step ensures symmetry and consistency at both ends of the material, affording even starting conditions for the middle part of the roll bending.

3) Middle roll bending: After both ends are pre-bent, the next step is the middle part of the roll bending operation, which is the most critical part of the entire rolling process. This stage involves the coordinated operation of all four rollers to achieve continuous smooth bending of the material’s middle part by precisely controlling the movement of each roller.

4) Final formation and springback control: The last step is final formation, which is to complete the shape of the entire roll bending and springback control of the material. At this stage, by adjusting the roller’s pressure and position, the geometric accuracy of the material after forming can be carefully controlled. Moreover, the springback effect can be predicted and compensated to ensure that the final product’s shape aligns with the height required by the design.

To improve the accuracy and efficiency of the simulation, the entire roll bending forming model is meshed in detail. Special attention is paid to the use of finer meshes in areas where the curvature of the material changes significantly and where stress is expected to be concentrated. Such a meshing strategy helps capture subtle changes in the model in these critical areas, ensuring high precision in the simulation results. According to the process parameters determined in the above experiment, the operation process of the simulation is started. After the preliminary simulation results appear, the differences from the experimental results are carefully analyzed, and the parameters of the model are adjusted according to these differences.

## 4 Results and discussion

### 4.1 The contrast of stress-strain distribution in the roll bending

**Fig 4:**
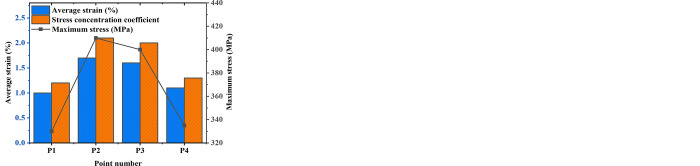
Stress-strain distributions at various roll bending speeds (a. 30 mm/s; b.45 mm/s; c.60 mm/s).

During the roll bending, the roll bending speed is adjusted to 30 mm/s, 45 mm/s, and 60 mm/s, and a comparative analysis of the stress-strain distribution is performed at different points. Fig 4 demonstrates a detailed comparison of the maximum stress, average strain, and stress concentration coefficient at central point P1, edge point P2, point P3 (near edge point), and P4 (far center point):

Fig 4 denotes that with the increase of roll bending speed, the maximum stress of the central point P1 and far center point P4 decreases slightly, while the average strain remains almost unchanged or declines slightly. It may be due to the thermal effect inside the material during the high-speed roll bending, which caused the material to soften, thus reducing the stress level. However, the maximum stress and average strain of the edge point P2 and near edge point P3 show the opposite trend, that is, the maximum stress increases and the average strain adds slightly. It indicates that the increase in roll bending speed may lead to intensified stress concentration in the edge region.

From the viewpoint of the stress concentration coefficient, all points’ stress concentration coefficient increases with the rise of roll bending speed, especially for P2 and P3. This further confirms that at higher roll bending speeds, the effect of stress concentration on the edge and its vicinity is more remarkable and may increase the risk of material failure, especially when performing roll bending forming processes requiring high accuracy or high strength.

Hence, it indicates that roll bending speed has an appreciable impact on the stress-strain distribution of the material, especially in the edge region. This phenomenon suggests that to optimize the rolling process, reduce stress concentration, and improve the quality of formed parts, careful control of roll bending speed is necessary, or determining the optimal combination of process parameters through prior simulation analysis. Different settings of roll bending speed reflect diverse material behavioral characteristics, and by comprehensively analyzing stress-strain data and stress concentration factors, a deeper understanding of the material’s mechanical response during roll bending can be achieved. This understanding helps achieve precise control of the rolling process in industrial applications, thereby improving the geometric accuracy and mechanical performance of finished products.

### 4.2 Changes in springback characteristics

**Fig 5:**
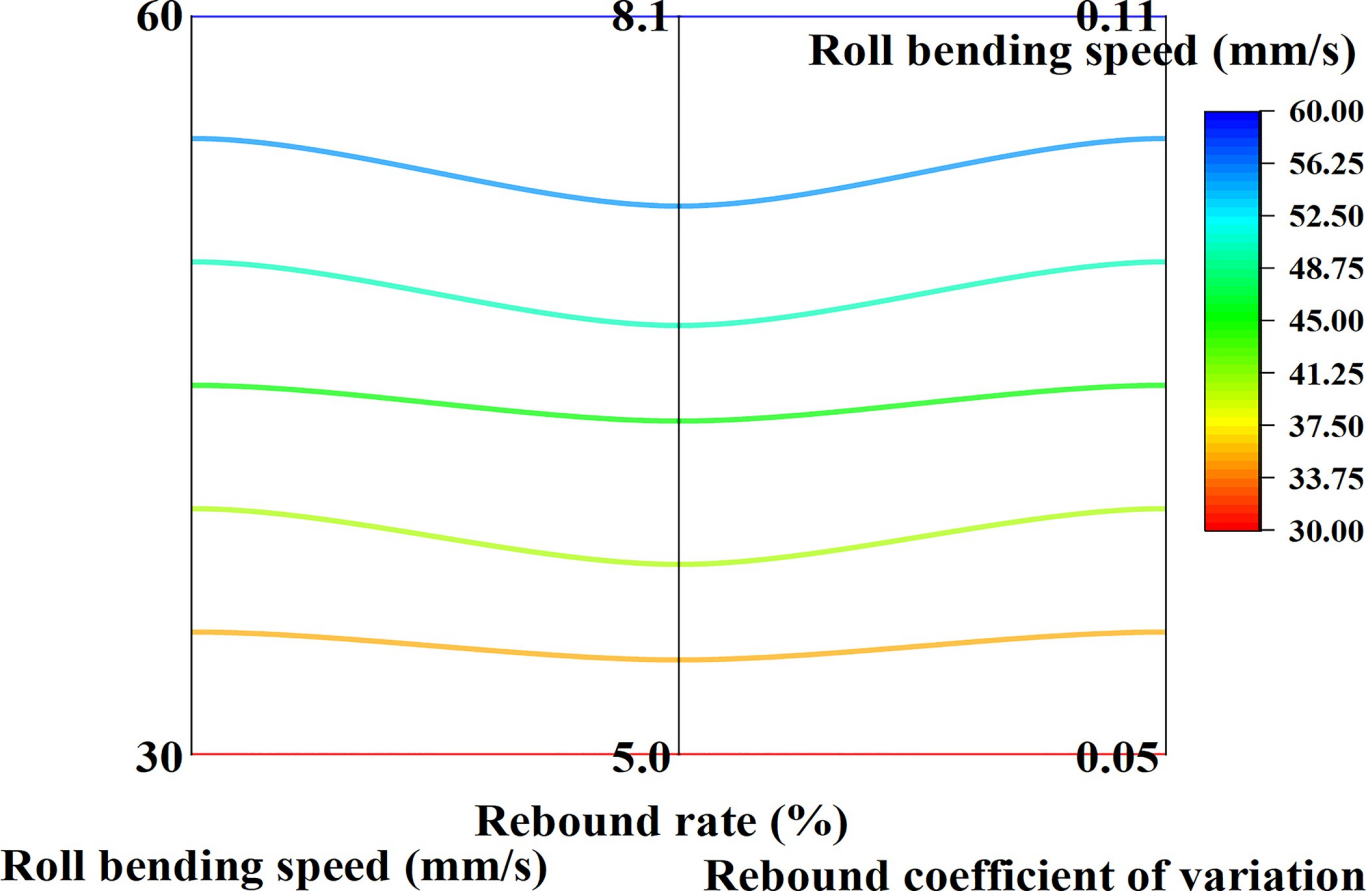
The relationship between roll bending speed and springback rate.

Fig 5 illustrates an approximate 3-percentage-point increase in the springback rate when the roll bending speed adds from 30mm/s to 60mm/s, revealing the impact of speed increase on the rise in the springback rate. It is also evident that there is a positive correlation between roll bending speed and springback rate. As the roll bending speed increases, so does the springback rate. This trend signifies that the material’s elasticity recovery capacity is enhanced with the rise in roll bending speed, making the formed part more prone to springback. Additionally, the gradual improvement in the variation coefficient of springback suggests that this phenomenon becomes more unstable with the increase in roll bending speed, possibly due to the temperature rise effect caused by high-speed roll bending or different adjustments in internal microstructures.

**Fig 6:**
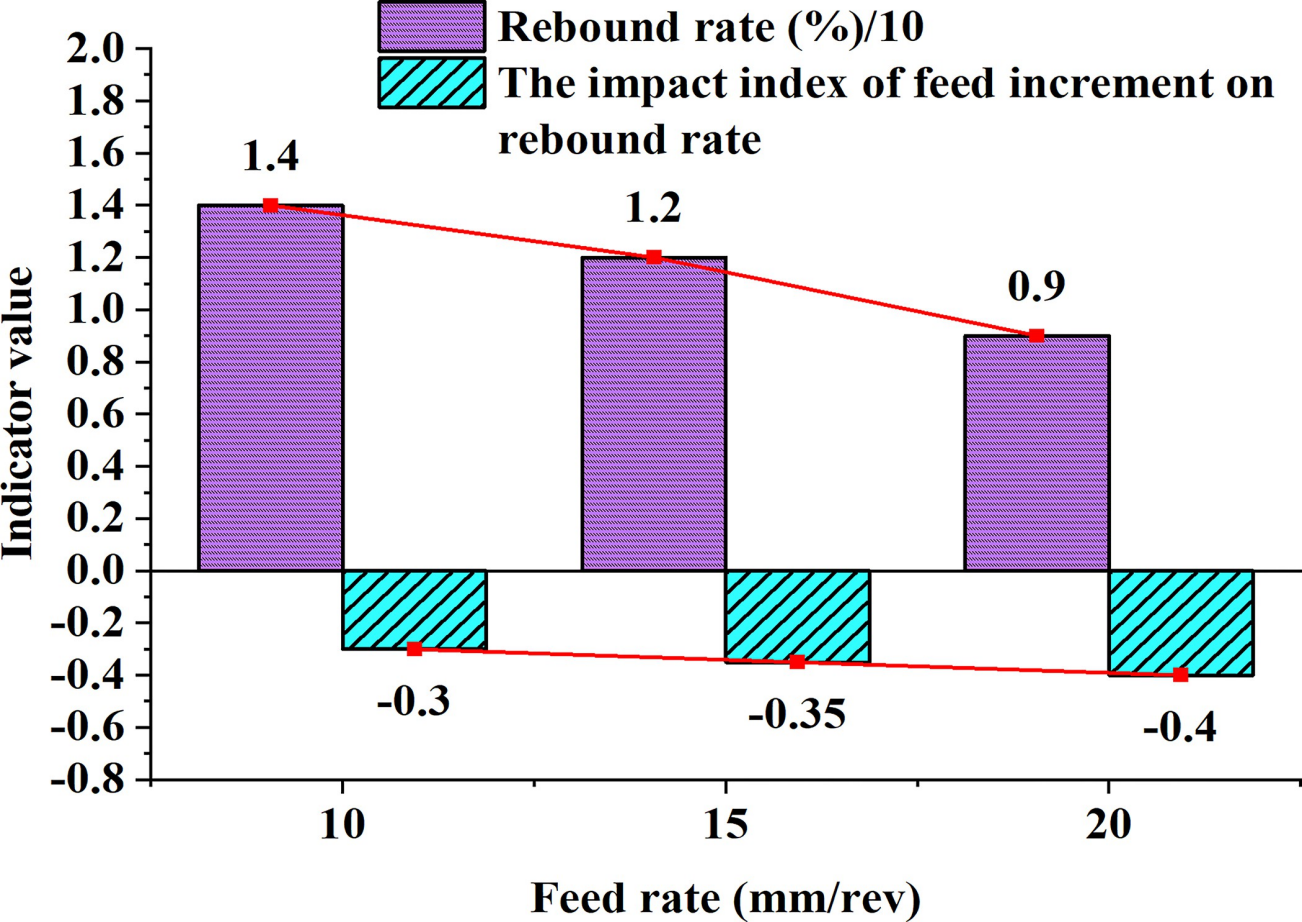
The correlation between feed rate and springback rate.

Further, the influence of feed rate on springback rate is studied, as plotted in Fig 6.

**Fig 7:**
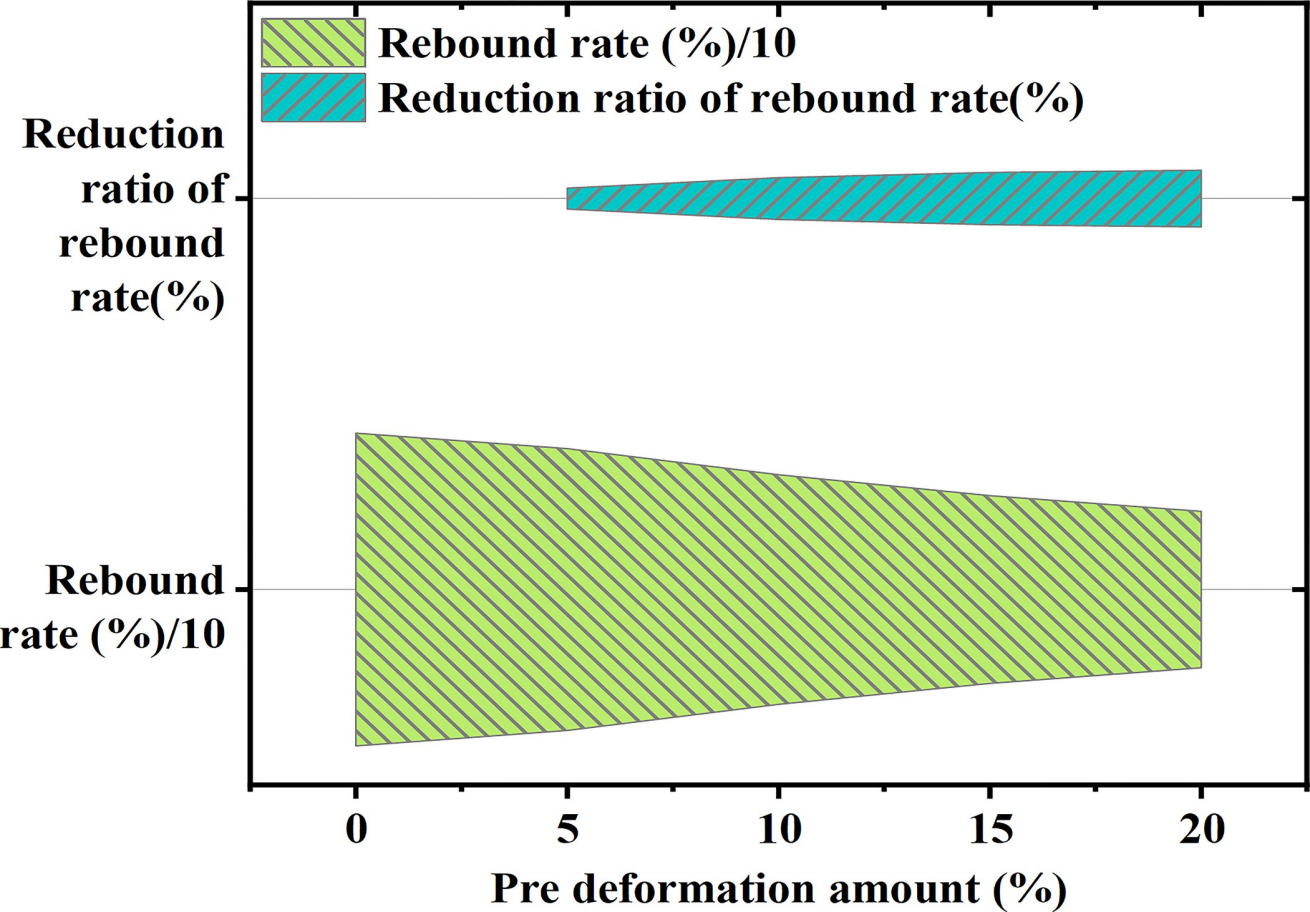
The trend of changes in pre- deformation amount and springback rate.

Fig 6 shows an overall decreasing trend in the springback rate with the increase in feed rate, and the exponential effect of feed increment on the springback rate is negative, indicating that increasing the feed rate can effectively reduce the springback rate. This may be because a larger feed rate subjects the material to greater plastic deformation during the forming process, thereby decreasing the amount of elastic recovery after forming. This phenomenon is crucial for rolling processes that require controlling springback to maintain product shape accuracy. A 20% increase in feed rate results in an average decrease in springback of approximately 7%. Consequently, increasing the roll bending speed exacerbates the springback phenomenon while improving the feed rate appropriately can mitigate springback to some extent. Changing trend of the pre-deformation amount and springback rate is suggested in Fig 7:

In Fig 7, with the increase in pre-deformation amount, the springback rate gradually decreases, and for each 1% increase in pre-deformation amount, the proportion of springback rate reduction also increases. This indicates that pre-deformation is an effective method to reduce springback in formed parts. The increase in pre-deformation amount means that the material has already undergone a certain degree of plastic deformation before roll bending, which diminishes the proportion of elastic deformation during the forming process, thus reducing the springback. This finding suggests that by appropriately pre-deforming the material beforehand, the springback rate of rolling products can be effectively reduced, providing a feasible technical approach to improving dimensional accuracy and decreasing subsequent correction work for finished products.

### 4.3 Comparison of experiment and simulation

**Fig 8:**
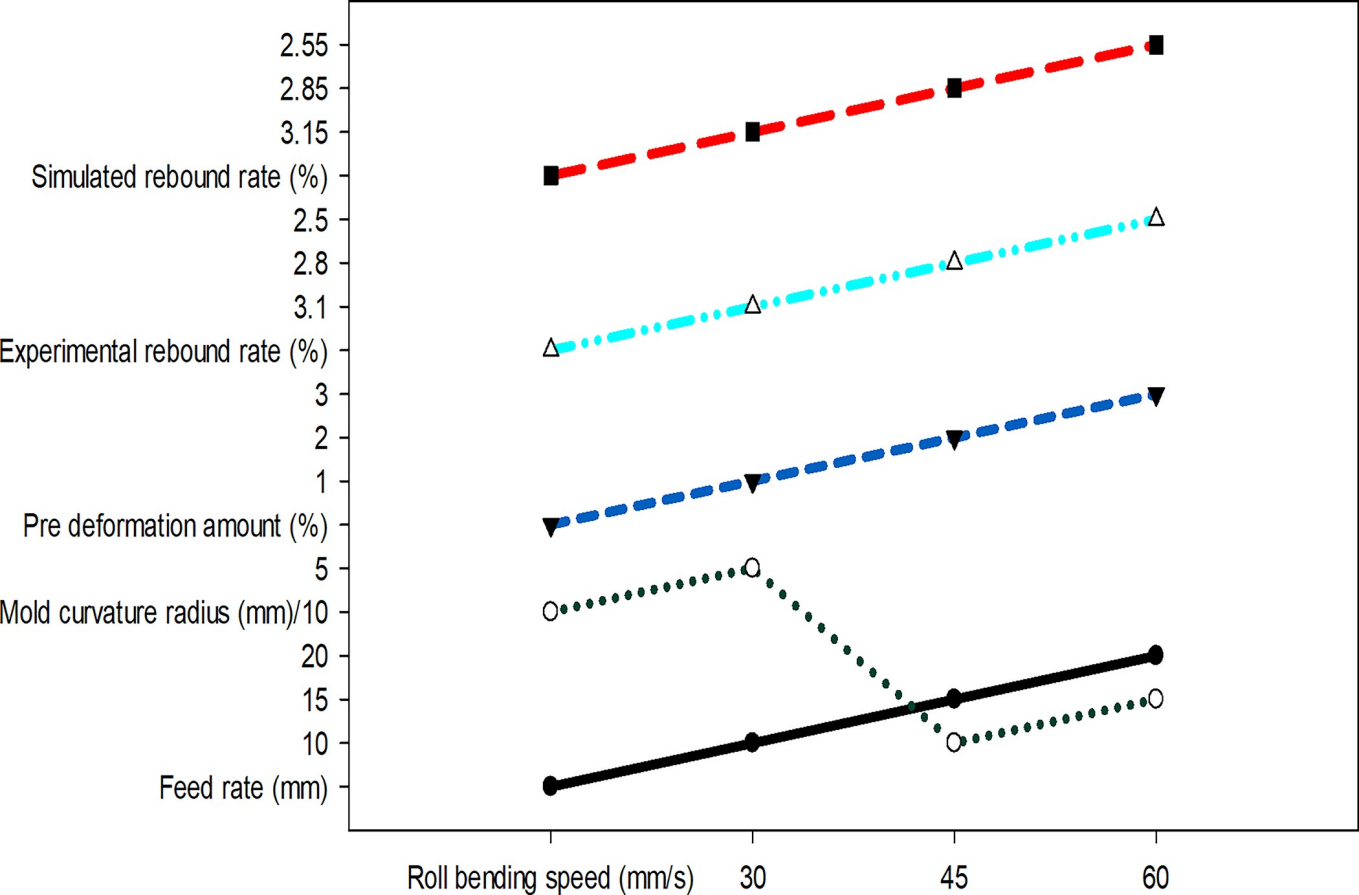
The influence of comprehensive process parameters on springback rate.

Fig 8 depicts the variations of springback rate in the experiment and simulation under different combinations of process parameters:

Fig 8 shows the influence of comprehensive process parameters on the springback rate, and reveals the effect trend of different process parameters on the springback rate of aluminum alloy after roll bending through the comparison of experimental and simulated data. In Fig 8, with the increase of roll bending speed, both experimental and simulated springback rates show a downward trend. This may be because the higher rolling speed leads to the higher temperature of the material in the forming process, which increases the plastic deformation ability of the material, thus reducing the springback phenomenon. Meanwhile, with the increase of feed rate, the rebound rate also shows a downward trend, which shows that the larger feed rate makes the material experience greater plastic deformation in the forming process, reducing the elastic recovery, thus reducing the rebound rate.

In addition, the increase of the curvature radius of the die is also related to the decrease of the springback rate. The larger radius of curvature of the die may reduce the bending degree of the formed part, thus reducing the elastic deformation ability of the material and further reducing the springback. The increase of pre-deformation also leads to the decrease of springback rate, which shows that proper pre-deformation of the material before rolling bending can effectively compensate the springback after forming, and the elastic recovery after forming can be reduced by pre-applied plastic deformation. By analyzing the data in Fig 8, the comprehensive effects of these process parameters on springback rate from multiple dimensions and the similarity between experimental and simulation results can be understood from multiple dimensions. As the roll bending speed increases from 30mm/s to 60mm/s, a decreasing trend is observed in both experimental and simulated springback rates. This may be attributed to the higher roll bending speed causing the material to attain a higher temperature during the forming process, thereby increasing its plastic deformation ability and reducing springback. A growth in the feed rate is accompanied by a decrease in the springback rate. This suggests that a higher feed rate results in more plastic deformation of the material, reducing its elastic recovery and consequently decreasing springback. With the mold curvature radius rising from 50mm to 150mm, both experimental and simulated springback rates exhibit a decreasing trend. This could be because a larger mold curvature radius reduces the bending degree of the formed part, thereby reducing the material’s elastic deformation ability and consequently decreasing springback. Similarly, an increase in the pre-deformation amount leads to a decrease in the springback rate. This illustrates that pre-deformation applied to the material before roll bending can effectively compensate for springback, reducing elastic recovery after forming through the plastic deformation applied in advance. The experimental springback rate is very close to the simulation springback rate, indicating that the simulation model can predict the actual springback behavior well. This high degree of consistency reveals that simulation technology can be used as an effective tool to evaluate and optimize process parameters before actual production, thereby reducing the cost and time of experiments.

## 5 Discussion

In the roll bending process of aluminum alloy materials, it is very important to accurately predict and control the springback phenomenon for improving product quality and reducing cost. This study focuses on the springback characteristics and its influence law of aluminum alloy four-axis flexible roll bending, which complements and deepens the related research in the existing literature. For example, Kut et al. (2021) [33] studied the springback prediction of aluminum alloy square pipe under pure moment. In this study, finite element method (FEM) is used to simulate the springback behavior of aluminum alloy square tube under pure bending moment, and the influence of different process parameters on springback is analyzed. The results show that the springback of aluminum alloy square tube can be effectively predicted and controlled by optimizing the magnitude of bending moment and the position of bending moment action point. This study provides theoretical basis and technological guidance for the precise forming of aluminum alloy pipes, and is of great significance for the application of aluminum alloy pipes in automobile, aerospace and other fields.

Engler et al. (2016) [34] studied the flexible roll bending process of aluminum alloy sheet, and optimized the process parameters and controlled the material properties. The effects of rolling speed, feed rate and friction conditions on the formability of aluminum alloy sheet were studied by combining experiments and numerical simulation. The results show that the forming quality of aluminum alloy sheet can be significantly improved and the defects can be reduced by reasonable selection of process parameters. This study provides an optimization strategy for the flexible roll bending process of aluminum alloy sheet, and has important reference value for improving the forming accuracy of aluminum alloy sheet and reducing the production cost.

Aday (2019) [35] used FEM to analyze the springback behavior of steel and aluminum alloy sheets. In this study, the finite element models of steel and aluminum alloy sheets are established, and the springback process under different thicknesses, different material properties and different bending moments is simulated. By comparing the experimental data with the simulation results, the accuracy of the finite element model is verified. The results show that the finite element method can effectively predict the springback behavior of different materials and provide an effective tool for the optimization of sheet metal forming process. This research has important engineering application value for improving sheet metal forming accuracy and reducing production cost.

To sum up, these literature provide valuable theoretical basis and technical reference for this study. On this basis, this study further discusses the influence of key process parameters in four-axis flexible roll-bending forming on the springback characteristics of aluminum alloy. Through the method of combining experiment and simulation, the comprehensive effects of rolling speed, die curvature radius, feed rate and pre-deformation are deeply analyzed, which provides a more comprehensive theoretical basis and practical guidance for the optimization of aluminum alloy roll-bending forming process and springback control.

## 6 Conclusion

In this study, the springback characteristics and its influence law in the process of four-axis flexible roll bending of aluminum alloy are deeply discussed, and a series of innovative results are obtained. By combining experiments with finite element simulation, the effects of key process parameters such as rolling speed, die curvature radius, feed rate and pre-deformation on the springback behavior of aluminum alloy are systematically analyzed. It is found that these process parameters can significantly affect the springback rate, among which increasing the rolling speed, feeding rate and pre-deformation can effectively reduce the springback rate, and increasing the curvature radius of the die can also help reduce the springback phenomenon. In addition, the high consistency between the experimental and simulation results verifies the accuracy of the simulation model and provides a reliable theoretical basis for the optimization of process parameters in actual production. The research results show that the springback can be effectively predicted and compensated by accurately controlling the process parameters, and the dimensional accuracy and shape quality of the formed parts can be improved. This is of great engineering value and practical significance for the application of aluminum alloy materials in high-end fields such as aerospace and automobile manufacturing. Meanwhile, this study also provides a new perspective and methodological guidance for the further research and industrial application of aluminum alloy roll bending process. The future work will continue to explore more factors affecting springback, such as roller gap and working temperature, and introduce more complex material models to simulate and control the springback phenomenon in the actual rolling and bending process more comprehensively. However, the material model used in the simulation may not fully capture the behavior of the actual material under complex stress states, especially regarding nonlinear large deformation and material damage. Although this study considers major process parameters, other factors such as roller clearance and temperature are not taken into account, which may also affect the springback phenomenon. Hence, plans include introducing more complex and accurate material models to better simulate the real behavior of materials during the rolling process, particularly considering the temperature dependence and damage evolution of materials. Additionally, further research into other process parameters that may affect springback during roll bending, such as roller clearance and working temperature, is essential for a comprehensive understanding and control of the springback phenomenon in roll bending.

## Supporting information

S1 File (ZIP)
