# Supplementary material for: Springback characteristics and influencing laws of four-axis flexible roll bending forming for aluminum alloy
Source: PLoS One. 2024 Aug 27;19(8):e0306604. doi: 10.1371/journal.pone.0306604 (PMC11349108; doi:10.1371/journal.pone.0306604)
Supplement: S1 File — (ZIP) [file pone.0306604.s001.zip › Data packet/code description.docx]

Code explanation:

Import the necessary libraries:

numpy is used to do mathematical calculations.

matplotlib.pyplot is utilized to display the results in plots.

scipy.optimize.curve_fit is employed to fit the data.

Simulation function complex_rebound_model:

This function accepts roll bending speed, feed rate, mold curvature radius, pre-deformation amount, and temperature as input parameters.

The complex interactions and nonlinear effects between these variables are simulated inside the function, especially the temperature of the nonlinear effect.

Generate simulated data:

numpy.random.uniform is employed to generate random data for different process parameters to simulate the diversity of actual production conditions.

Calculated springback rate:

For each set of generated process parameters, the complex_rebound_model function is used to calculate the corresponding rebound rate.

Data analysis example: roll bending speed influence on springback rate:

The curve_fit function is used for curve fitting of the relationship between roll bending speed and springback rate.

Actual data points and the fitting curve are drawn to intuitively show the relationship between them.

Operating environment:

Python version: It is recommended to use Python 3.6 and above to ensure that all libraries work properly.

Necessary Python Libraries:

numpy: numerical calculations are performed.

matplotlib: It is used to plot graphs.

scipy: It provides advanced mathematical tools such as curve fitting.

Installation Command:

The required libraries can be installed using the pip command:

bash

Copy code

pip install numpy matplotlib scipy

Operation mode:

the above code is saved as a. Py files, such as simulation_example. Py. Then, on the command line or terminal, it switches to the directory where the file is located and runs the following command:

bash

Copy code

python simulation_example.py

Once executed, scatter plots and fitted curves of the relationship between roll speed and springback rate can be seen.
